# mTORC1 Signaling Pathway Mediates Chronic Stress-Induced Synapse Loss in the Hippocampus

**DOI:** 10.3389/fphar.2021.801234

**Published:** 2021-12-20

**Authors:** Yu-Fei Luo, Xiao-Xia Ye, Ying-Zhao Fang, Meng-Die Li, Zhi-Xuan Xia, Jian-Min Liu, Xiao-Shan Lin, Zhen Huang, Xiao-Qian Zhu, Jun-Jie Huang, Dong-Lin Tan, Yu-Fei Zhang, Hai-Ping Liu, Jun Zhou, Zu-Cheng Shen

**Affiliations:** ^1^ Department of Pharmacology, School of Pharmacy, Fujian Medical University, Fuzhou, China; ^2^ Clinical Medical Research Center, Hunan Prevention and Treatment Institute for Occupational Diseases, Changsha, China; ^3^ Department of Pharmacology, School of Basic Medicine and Life Science, Hainan Medical University, Haikou, China; ^4^ Department of Pharmacy, Wuhan No. 1 Hospital, Wuhan, China; ^5^ Department of Pharmacology, School of Basic Medicine, Tongji Medical College, Huazhong University of Science and Technology, Wuhan, China; ^6^ Translational Medicine Center, Xi’an Chest Hospital, Medical College of Xi’an Jiaotong University, Xi’an, China

**Keywords:** chronic restraint stress, mammalian target of rapamycin, postsynaptic density protein 95, depression, fluoxetine

## Abstract

**Background:** The mechanistic target of rapamycin complex 1 (mTORC1) signaling has served as a promising target for therapeutic intervention of major depressive disorder (MDD), but the mTORC1 signaling underlying MDD has not been well elucidated. In the present study, we investigated whether mTORC1 signaling pathway mediates synapse loss induced by chronic stress in the hippocampus.

**Methods:** Chronic restraint stress-induced depression-like behaviors were tested by behavior tests (sucrose preference test, forced swim test and tail suspension test). Synaptic proteins and alternations of phosphorylation levels of mTORC1 signaling-associated molecules were measured using Western blotting. In addition, mRNA changes of immediate early genes (IEGs) and glutamate receptors were measured by RT-PCR. Rapamycin was used to explore the role of mTORC1 signaling in the antidepressant effects of fluoxetine.

**Results:** After successfully establishing the chronic restraint stress paradigm, we observed that the mRNA levels of some IEGs were significantly changed, indicating the activation of neurons and protein synthesis alterations. Then, there was a significant downregulation of glutamate receptors and postsynaptic density protein 95 at protein and mRNA levels. Additionally, synaptic fractionation assay revealed that chronic stress induced synapse loss in the dorsal and ventral hippocampus. Furthermore, these effects were associated with the mTORC1 signaling pathway-mediated protein synthesis, and subsequently the phosphorylation of associated downstream signaling targets was reduced after chronic stress. Finally, we found that intracerebroventricular infusion of rapamycin simulated depression-like behavior and also blocked the antidepressant effects of fluoxetine.

**Conclusion:** Overall, our study suggests that mTORC1 signaling pathway plays a critical role in mediating synapse loss induced by chronic stress, and has part in the behavioral effects of antidepressant treatment.

## Introduction

Major depressive disorder (MDD) is one of the most common neuropsychiatric disorders characterized by low mood, hopelessness and suicidality, which seriously threatens human health and leads to heavy socioeconomic burdens (Simon, 2003; Armbrecht et al., 2021). The primary drug therapy of MDD is only effective in 40–70% of MDD patients (Little, 2009; Papakostas and Ionescu, 2015). To overcome these drawbacks, it was urgently necessary to clarify pathophysiological mechanisms underlying the development of depression and find new therapeutic targets.

Previous studies have shown that hippocampal volume reduction and synaptic deficits in functional connectivity are hypothesized to contribute to symptoms associated with MDD (Bremner et al., 1995; Admon et al., 2013); Moreover, losses of spine number and dendritic arbor in hippocampus were observed in mice exposed to chronic stress (Conrad et al., 2012), these findings suggest that neuronal atrophy in hippocampus was associated with MDD and depression-like behaviors. Additionally, ketamine, an N-methyl-D-aspartate receptor antagonist, is the most prominent antidepressant for treatment-resistant depression, and rapid improvement of hippocampal dendritic atrophy, attenuation of postsynaptic dendritic spine loss and increased neurogenesis were observed after ketamine therapy (Murrough, 2012; Krystal et al., 2017). However, the possible mechanisms underlying synaptic atrophy induced by chronic stress need to be ascertained.

Recently, evidence points toward that the mechanistic target of rapamycin complex 1 (mTORC1) signaling may be involved in regulating the translation and synthesis of synaptic proteins in prefrontal cortex and mediating depressive behaviors (Dwyer et al., 2015). An increasing evidence shown that mTORC1 signaling possibly mediates a rapid and robust efficacy of ketamine (Welberg, 2010; Yang et al., 2018), and some antidepressant drugs have differential effects on mTORC1 signaling in the rat hippocampal neurons (Park et al., 2014). However, the roles of mTORC1 signaling in the hippocampus in depression, as well as in the action of classic antidepressant drugs, are still unknown.

In the present research, we raised a hypothesis that mTORC1 signaling pathway plays critical roles in mediating hippocampal synapse loss and functional impairment induced by chronic stress, and is involved in the behavior-regulating effects of antidepressant drugs. We employed a mouse CRS model to induce depression-like behaviors in mice, and used this model to investigate the synapse loss and functional impairment in different regions of the hippocampus under the condition of chronic stress. The findings proved that mTORC1 signaling is impaired by long-term stress, and this further mediates synapse loss by suppressing synaptic protein synthesis. Importantly, mTORC1 signaling also participates in the antidepressant effects of fluoxetine. This study therefore provides mechanistic insights for the molecular tuning factor of mTORC1 signaling that regulates synaptic function and the therapeutic targets for major depression disorders, and also indicates that compounds which up-regulate mTORC1 signaling in the hippocampus may be effective for the treatment of depression.

## Materials and Methods

### Animals

In order to eliminate the effect of estrogen on mood, male mice were used in our research, and C57BL/6 male mice (20 ± 2 g, aged 7–8 weeks) were purchased from Shanghai Laboratory Animal Center (Shanghai, China; certificate number: SCXK2017-0005). Animals were housed in a specific-pathogen free animal facility and maintained at 24 ± 2°C under a 12 h light/12 h dark cycle with free access to food and water. The Institutional Animal Care and Use Committee of Fujian Medical University approved all the experimental protocols concerning the handling of mice.

### Agents

Rapamycin, fluoxetine and Triton X-100 were purchased from Sigma (St. Louis, MO, United States). BSA, HEPES and Tween-20 were obtained from Beijing Dingguo Changsheng Biotechnology Co., Ltd (Beijing, China). Other general agents were obtained from commercial suppliers. Fluoxetine (20 mg/kg) was freshly dissolved in saline and administered intraperitoneally once daily for 14 days. Rapamycin was prepared as a stock solution in DMSO (0.1 M), and its intracerebroventricular injection was set at the final concentration of 10 mg/ml and a volume of 2 μl once a day for each mouse.

### Chronic Restraint Stress Procedures

The chronic restraint stress (CRS) procedures used in this study was similar to the treatments described previously (Xu et al., 2017; Shen et al., 2019) with minor modification. After a one-week acclimatization period, mice were individually placed head-first into a well-ventilated 50 ml syringe tube and plugged. Mice were not able to move forward or backward in this device. This restraint stress was delivered to animals at set times daily from 11:30 to 15:30. Control mice remained in their original cages and were left undisturbed in this home environment. After restraint stress treatment, the restrained animals were returned to their normal home environment by housing them alone. This procedure was repeated for 21 days unless otherwise indicated.

### Behavioral Assessments

Behavioral analyses were performed by following the sequences depicted as below, and behavioral assessments were carried out using a computerized video-tracking system (SMART, Panlab S.I., Barcelona, Spain) by tracking mouse behavior. Mice were placed in the same environment 30 min prior to behavioral testing.

### Sucrose Preference Test

Mice were presented with two bottles: one filled with drinking water and the other with 1% sucrose solution (50 ml each). After 12 h of adaptation on the first day, the position of the sucrose bottle and water bottle was interchanged on the second day, and continuing to acclimate for another 12 h to avoid the formation of special position preference during drinking. On the second day, all the tested mice were water-deprived for 12 h, and the test was performed for 2 h on the third day. Consumption of water and sucrose solution was calculated by subtracting the remained weight of the bottles. Sucrose preference (%) was calculated as consumption of sucrose solution divided by total fluid consumption (water plus sucrose).

### Forced Swim Test

Mice were introduced into a plastic cylinder (40 cm deep, 20 cm in diameter) filled with water at 23–25°C up to a height of 25 cm from the bottom, and forced to swim for 6 min. The last 5-min swim session was recorded. After each swim session, the mouse was removed from the cylinder, dried with paper towels, placed in a resting cage for 20 min, and returned to its home cage. Water in the cylinder was renewed between each mouse. Mice were thought to be floating when there was no obvious swimming movement in the cylinder, and the time spent floating was recorded by the SMART software.

### Tail Suspension Test

Mice were allowed to adapt to the test room for at least 2 h prior to testing. In brief, cleaned tails of mice were wrapped in adhesive tape at approximately 1.5 cm from the tip. An experimental clip was attached to the adhesive tape. Mice were suspended 20–25 cm above the floor by the clip and were videotaped for 5.5 min by the SMART software. Immobility time in the last 5 min was measured from the recorded video. A mouse was considered immobile when it made no active escape movements but included passive swaying. A mouse was considered mobile when making running motions, body jerks, or attempting to catch its tail.

### Samples Collection

After behavioral tests, mice were anesthetized with isoflurane and quick decapitated, and the brains were moved into the ice-cold oxygenated artificial cerebrospinal fluid (aCSF). Coronal bilateral slices (300 μm in thickness) containing the hippocampus were cut by a vibratome (VT 1200S, Leica, Wetzlar, Germany) in the aCSF. According to the mouse Brain in Stereotaxic Coordinates, the first few slices containing the hippocampus were dorsal hippocampus and the last few slices containing the hippocampus were ventral hippocampus, while the middle slices is not easy to differentiate and ignored. After dorsal and ventral hippocampus differentiation, the slices were microdissected into CA1, DG and CA3 under the microscope. Compositions of aCSF (in mM): 119.0 NaCl, 1.3 MgSO_4_, 3.5 KCl, 1.0 NaH_2_PO_4_, 26.2 NaHCO_3_, 2.5 CaCl_2_ and 11.0 glucose, pH 7.4 (300 mOsm).

### Western Blotting Analysis

Total protein from the tissues was collected and homogenized in RIPA buffer (P0013B, Beyotime, Shanghai, China) with a cocktail of protease (Roche, Basel, Switzerland) and phosphatase inhibitors (Sigma-Aldrich, St. Louis, MO, United States). The protein concentration was measured using a Bradford protein assay kit (P0006C, Beyotime). Equal amounts (30 μg) of proteins were separated by 10% SDS-PAGE and then transferred to a nitrocellulose membrane. The blots were then blocked with 5% (w/v) BSA in Tris-buffered saline containing 0.1% Tween-20 (TBST) buffer for 1 h at room temperature and incubated with the corresponding primary antibodies overnight at 4°C. Subsequently, the membrane was washed three times with TBST and incubated with horseradish peroxidase (HRP)-conjugated secondary antibodies for 1 h at room temperature. Finally, the membrane was washed and visualized with the BeyoECL Star reagent (P0018AM, Beyotime). The relative optical densities of the bands were quantified using ImageJ gel analysis software. The band intensities were quantified and normalized to the corresponding controls. The detailed information about antibodies used was described in the Supplementary Table S1. All full original images of Western blotting assays for figure were shown in the Supplementary Material.

### RNA Isolation and Quantitative RT-PCR

RNAiso Plus reagent kit (Takara, Kyoto, Japan) was used to extract total RNA according to the manufacture’s requirements. Total RNA was reverse transcribed to cDNA using a Takara Prime Script RT reagent kit (RR047A, Takara, Kyoto, Japan). RT-qPCR was performed using a TB Green PCR kit (RR420A, Takara) with a QuantStudio 6 Flex Real-Time PCR System. The GAPDH gene was used as an endogenous control. The relative gene expression levels were calculated using the 2^−ΔΔCT^ method. The primer sequences used in this study were described in the Supplementary Table S2.

### Synaptic Fractionation

Samples were homogenized by sonication with ice-cold TEVP buffer (in mM) (0.32 sucrose, 10 Tris-HCl, pH 7.4, 5 NaF, 1 Na_3_VO_4_, 1 EDTA, 1 EGTA, 1% cocktail of protease inhibitor and phosphatase inhibitor). The homogenate was centrifuged at 1,000 g for 10 min at 4°C to obtain the nuclei, large debris and the supernatant, and then the supernatant was centrifuged at 10,000 g for 30 min at 4°C to achieve a crude synaptosomal fraction and the supernatant. The crude synaptosomal fraction was separated by 0.85/1.0/1.2 M sucrose density gradient centrifugation at 100,000 *g* for 2 h. The synaptosomes were obtained from the 1.0/1.2 M sucrose interface. After being washed with 0.5% Triton X-100, synaptosomal pellets were collected by centrifugation at 1,000 *g* for 10 min and then subjected to a second 1.0/1.5/2.0 M sucrose density gradient centrifugation at 100,000 *g* for 2 h. The postsynaptic density (PSD) fraction was obtained from the 1.5/2.0 M interface of the sucrose gradients. The PSD fraction was diluted with an equal volume of 1% Triton X-100/150 mM KCl solution, mixed for 5 min, and centrifuged at 201,800 g for 1 h. The resultant PSD pellet was resuspended in a 0.5% Triton X-100/75 mM KCl solution containing sodium orthovanadate and protease inhibitors. All experimental steps were performed at 4°C.

### Stereotaxic Injections

The implantation technique was performed as previously described (Xu et al., 2017). For intracerebroventricular injection, mice anesthetized with isoflurane (Shenzhen RWD Life Science) were mounted in an automated stereotactic aperture (RWD68001, Shenzhen RWD), and one stainless steel cannulas with 15 mm length and 0.6 mm outside diameter was implanted into the intracerebroventricular region (AP = −2.0 mm; ML = ± 1.5 mm; DV = −2.6 mm). The incision was closed with disposable sterile sutures and needles, and then carefully observed the conditions of the mice. The mice were placed back in their original cages after recovery from the surgical procedure. Drug injection volume and rate were controlled by an injection pump (RWD 68606, Shenzhen RWD). The microsyringe was left in place for an additional 2 min before both injection and withdrawal.

### Statistical Analysis

The data are presented as “mean ± S.E.M.”. The normality of the distribution of the data and the homogeneity of their variances were assessed, and one-way analysis of variance (ANOVA) with Bonferroni analysis was then used to compare the means from the different groups by using SPSS 18.0 software (SPSS Inc., Chicago, IL, United States). The differences between groups were considered statistically significant if *p* < 0.05.

## Results

### CRS Induces Depression-like Behaviors in Mice and Alters Hippocampal IEGs Expression

**FIGURE 1:**
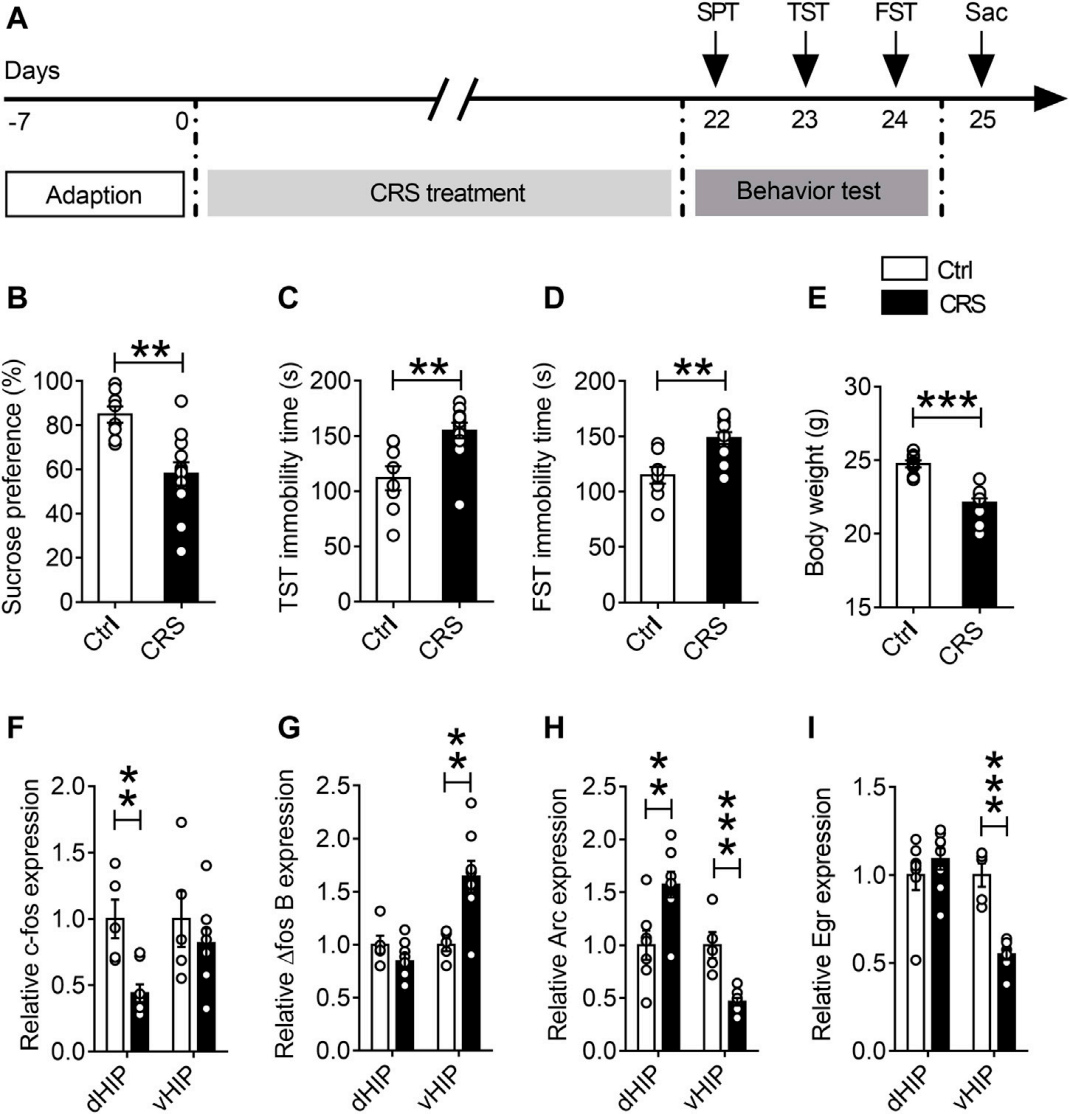
Effects of CRS on dorsal and ventral hippocampal IEG expression. **(A)** The schematic timeline of the experimental procedures. **(B–E)** Mice that exposed to CRS for 21 days displayed depression-like behavior, including reduced sucrose preference in the SPT **(B)**, increased immobile time in the TST **(C)**, increased floating time in the FST **(D)** and weight loss compared with the controls **(E)**. **(F–I)** RT-qPCR assays found that the mRNA level of *c-fos* was selectively decreased in the dHIP, and not significantly changed in the vHIP **(F)**. The mRNA level of △*fos B* was increased in the vHIP, without significant alteration in the dHIP **(G)**. The mRNA level of *Arc* was upregulated in the dHIP but downregulated in the vHIP **(H)**. The mRNA level of *Egr* was reduced in both the dHIP and vHIP **(I)**. Data are presented as mean ± SEM. One-way ANOVA for all. n = 8-12 per group for **(B–E)**; *n* = 5–7 per group for **(F–I)**. **p < 0.01, ***p < 0.001 vs. Ctrl group.

Mice were immobilized in the restrainer once for 4 h daily for 21 consecutive days. At the end of CRS modeling, depression-like behaviors were assessed by the SPT, TST, and FST tests (Figure 1A). The results showed that, compared with the control group, mice that underwent the CRS had a reduced sucrose preference in SPT (*n* = 8–12, *p* = 0.0014) (Figure 1B). Moreover, CRS increased the immobility time both in TST (*n* = 8–12, *p* = 0.0025) (Figure 1C) and FST (*n* = 8–12, *p* = 0.0014) (Figure 1D). Besides, a significant lowness in weight was observed in the CRS group when compared with the control group (*n* = 8–12, *p* < 0.0001) (Figure 1E). These results suggest that the mice developed depression-like behaviors after undergoing restraint stress for 4 h a day for 21 consecutive days.

Since many IEGs are rapidly and robustly activated after sensory and behavioral experience, and are believed to be critical for converting experience into long-term memory (Sun and Lin, 2016), we used qPCR to detect the expression of IEGs in dorsal hippocampus (dHIP) and ventral hippocampus (vHIP) after chronic stress exposure, including *c-fos*, *Arc* and *Egr*. The results displayed that the expression of *c-fos* gene was selectively decreased in dHIP (*n* = 5–7, *p* = 0.0031), but there was no significant difference in vHIP (Figure 1F). Interestingly, CRS only increased the mRNA levels of △*fos B* in vHIP, but had no effect on △*fos B* in dHIP (Figure 1G). Moreover, we found that mRNA level of *Arc* increased in dHIP (*n* = 5–7, *p* = 0.0023) and decreased in vHIP of CRS mice (*n* = 5–7, *p* < 0.0001) (Figure 1H). Additionally, CRS observably reduced the mRNA expression of *Egr* gene in vHIP (*n* = 5–7, *p* < 0.0001) (Figure 1I). These findings strongly suggest that CRS alters neuronal activity in the hippocampus.

### CRS Alters Glutamate Receptors Expression in the Hippocampus of Mice

**FIGURE 2:**
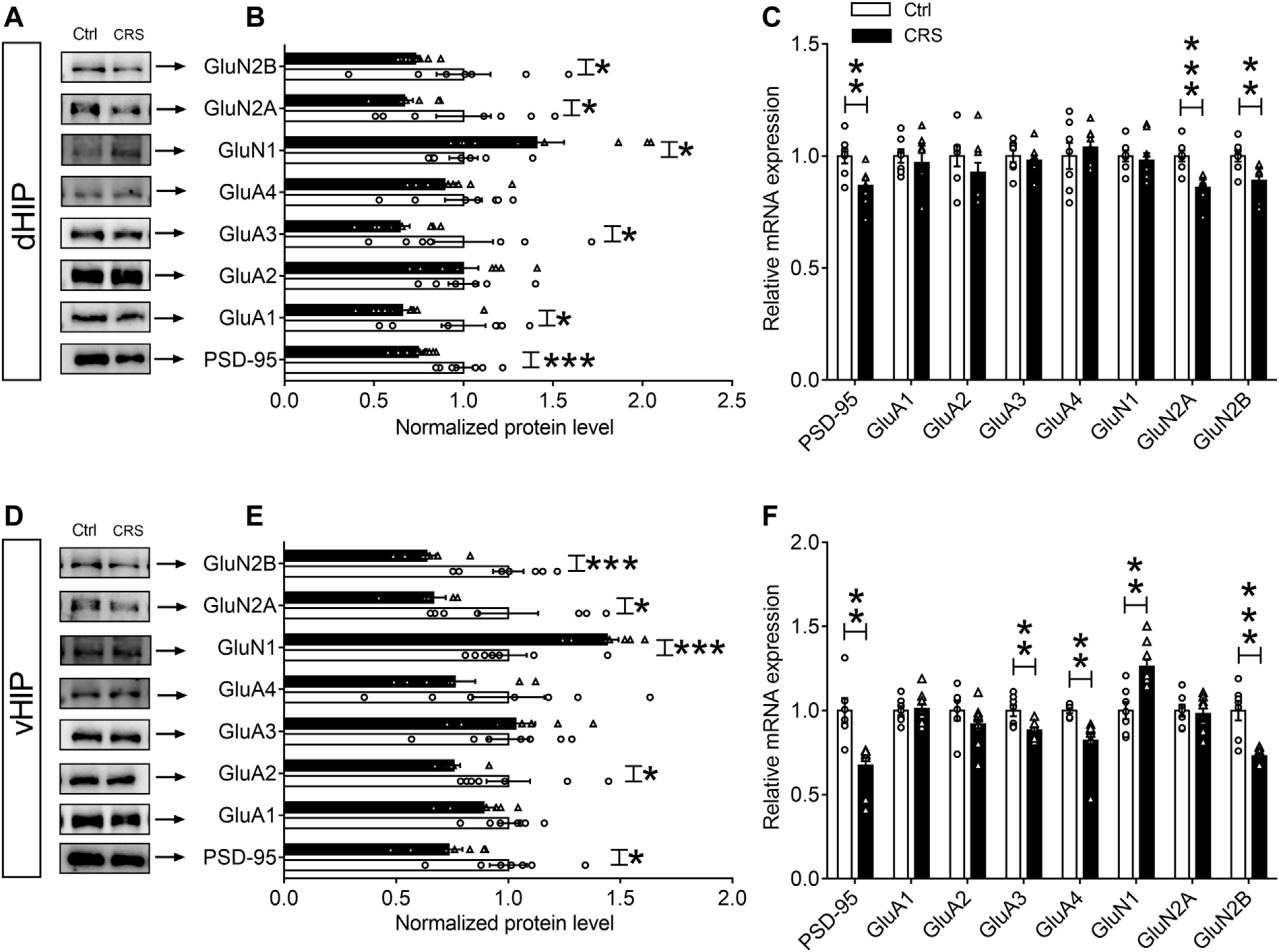
Expression of glutamate receptors in the hippocampus of chronically stressed mice. **(A,B)** CRS selectively decreased the expression levels of PSD-95, GluA1, GluA3, GluN2A and GluN2B, while increased GluN1 expression in the dHIP. **(C)** The mRNA levels of PSD-95, GluN1 and GluN2A were reduced in the dHIP after CRS. **(D,E)** CRS selectively decreased the expression of PSD-95, GluA2, GluN2A and GluN2B, while increased GluN1 expression in the vHIP. **(F)** The mRNA levels of PSD-95, GluA3, GluA4, and GluN2B were reduced; while GluN1 mRNA level was upregulated in the vHIP after CRS. Data are presented as mean ± SEM. One-way ANOVA for all. *n* = 7–9 per group for **(B,C,E,F)**. *p < 0.05, **p < 0.01, ***p < 0.001 vs. Ctrl group.

Evidence has indicated that expression of c-Fos and Arc reflects the activity of neurons and the induction of activity-associated plasticity in neurons (Josselyn et al., 2015; Holtmaat and Caroni, 2016). To address whether changes in neuronal activity are due to synaptic damage in CRS-induced depression-like behaviors, Western blotting and qPCR assays were used to measure the levels of NMDARs (GluN1, GluN2A and GluN2B) and AMPARs (GluA1, GluA2, GluA3 and GluA4) in dHIP and vHIP. The results of Western blotting showed that CRS remarkably decreased the expression of GluN2A and GluN2B subunits of NMDARs in both dHIP and vHIP (*n* = 7–9), but increased the expression of GluN1 (*n* = 7–9) (Figures 2A,B,D,E). However, the effect of CRS on AMPARs expression was different in dHIP and vHIP. CRS markedly reduced the expression of GluA1 and GluA3 subunits of AMPARs, and did not affect the expression of GluA2 and GluA4 in dHIP, while only decreased expression of GluA2 was observed in vHIP (*n* = 7–9). As shown in Figures 2C,F, the levels of mRNA were not consistent with the expression of proteins. In dHIP, CRS just decreased the mRNA levels of GluN1 and GluN2A of NMDARs after CRS exposure, and did not influence the mRNA levels of subunits of AMPARs. In vHIP, CRS reduced the mRNA levels of GluA3 and GluA4. Moreover, it was found that the mRNA levels of GluN2B decreased but the mRNA levels of GluN1 increased in vHIP of CRS mice. Especially, we found that the expression of PSD-95 protein and mRNA was significantly decreased in dHIP and vHIP of CRS mice. These data indicate that CRS-induced depression-like behaviors were associated with destruction of synapses in the hippocampus of mice.

**FIGURE 3:**
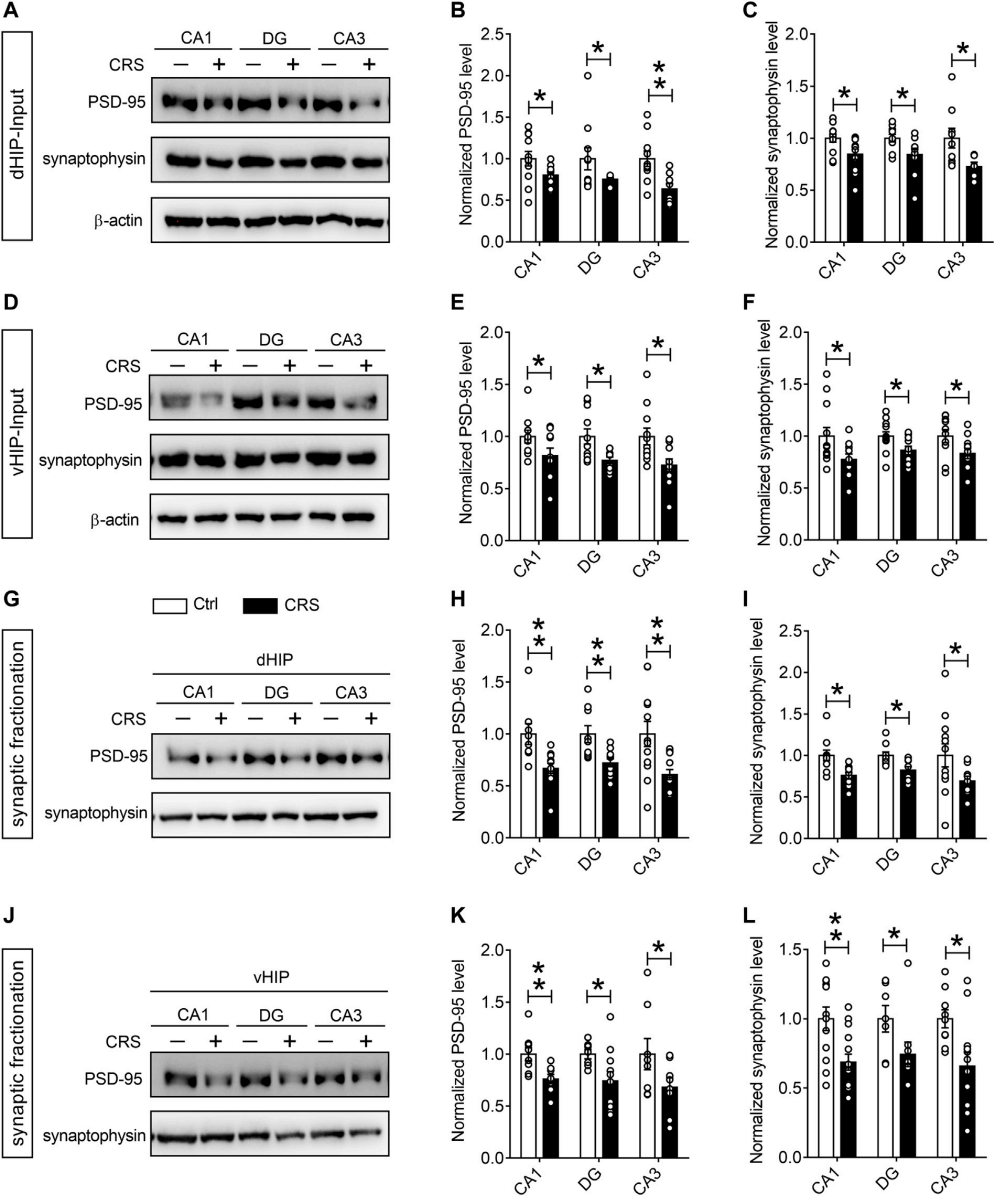
CRS mediates remarkable synapse losses in the hippocampus. **(A–C)** CRS decreased the expression of PSD-95 and synaptophysin in the CA1, DG, and CA3 regions of dHIP; representative Western blots **(A)**, statistical analysis of PSD-95 **(B)** and synaptophysin **(C)**. **(D–F)** CRS reduced the PSD-95 and synaptophysin expression in the CA1, DG, and CA3 of vHIP; representative Western blots **(D)**, statistical analysis of PSD-95 **(E)** and synaptophysin **(F)**. CRS induced synapse loss in the CA1, DG and CA3 of dHIP **(G–I)** and vHIP **(J–L)**; representative Western blots **(G, J)**, statistical analysis of PSD-95 **(H, K)** and synaptophysin **(I, L)**. Data are presented as mean ± SEM. One-way ANOVA for all. *n* = 10–9 per group for **(A–F)**, *n* = 8–9 per group for **(G–L)**. *p < 0.05, **p < 0.01 vs. Ctrl group.

For quantitative assessment of synapses, we next evaluated synaptic markers in dentate gyrus (DG), CA1 and CA3 regions of dHIP and vHIP, including synaptic vesicle proteins such as synaptophysin, and postsynaptic markers such as PSD-95 (Onwordi et al., 2020). The results of Western blotting showed that the expression of PSD-95 and synaptophysin in the CA1, DG and CA3 regions of dHIP (*n* = 10–9) (Figures 3A–C) and vHIP (*n* = 10–9) (Figures 3D–F) were significantly reduced after CRS exposure. We then asked whether CRS treatment could result in synaptic loss in the hippocampus, and detected the expression of PSD-95 and synaptophysin in the extracted synaptosome fractions from CA1, DG, and CA3 of dHIP and vHIP (*n* = 9–10). Results obtained from the analysis of synaptosome proteins were similar with the analysis results from total protein samples (Figures 3G–L). Taken together, the above results indicate that CRS-induced depression-like behaviors were accompanied by synaptic dysfunction in the hippocampus of mice.

### CRS Mediates Downregulation of Protein Synthesis by Targeting mTORC1 Signaling

**FIGURE 4:**
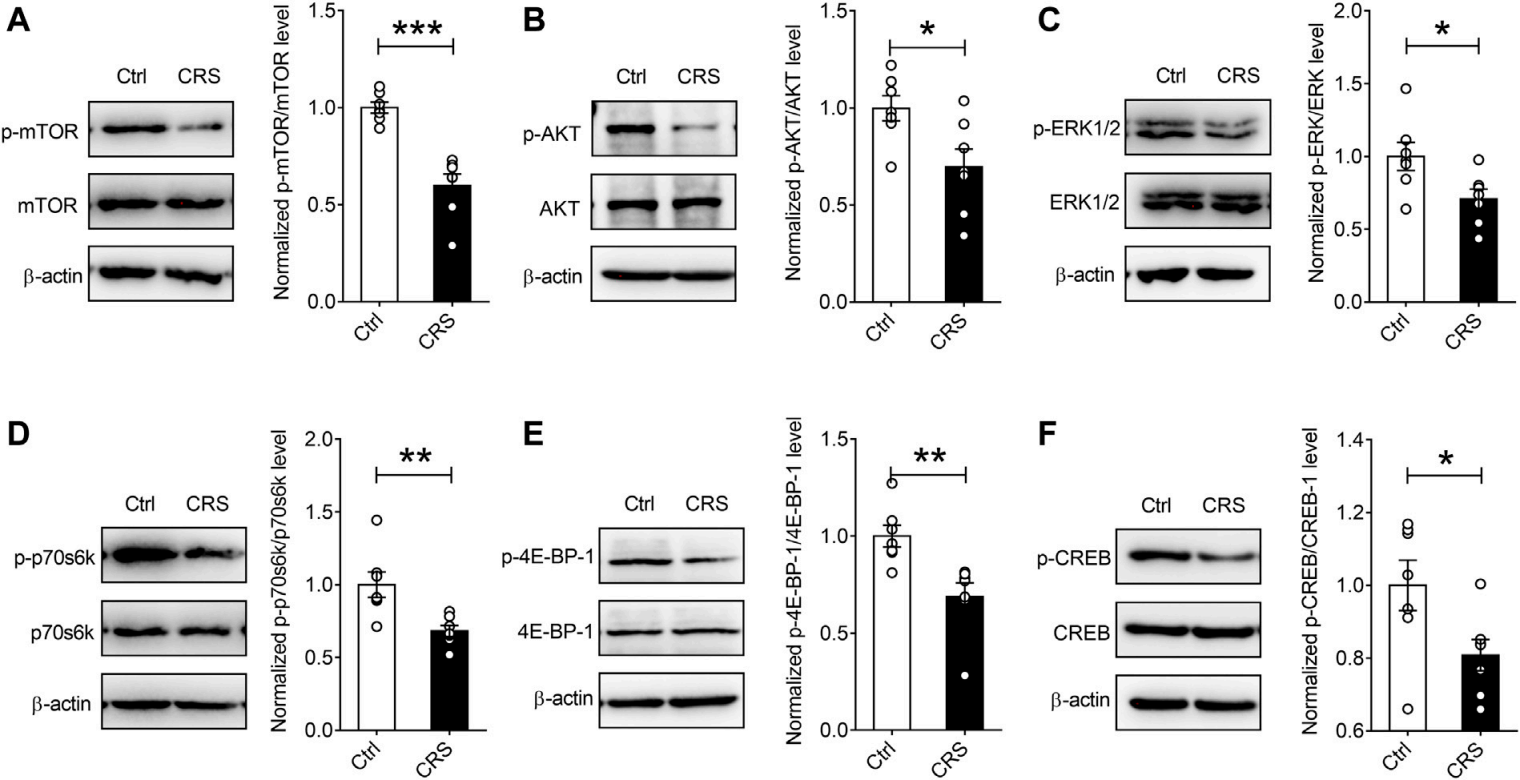
CRS mediates protein synthesis decreases by targeting mTORC1 signaling in the hippocampus. CRS significantly decreased the phosphorylation levels of the mTORC1 signaling pathway-associated targets, such as p-mTOR **(A)**, p-Akt **(B)**, p-ERK **(C)**, p-p70S6K **(D)**, p-4E-BP-1 **(E)** and p-CREB **(F)**; while no effects on the expression of total mTOR, Akt, ERK, p70S6K, 4E-BP-1 and CREB were observed. Data are presented as mean ± SEM. One-way ANOVA for all. *n* = 7 per group for all. *p < 0.05, **p < 0.01, ***p < 0.001 vs. Ctrl group.

Previous evidence indicates that one of the signaling pathways associated with protein synthesis-dependent synaptic plasticity is the mTORC1 signaling (Hou et al., 2017), and mTORC1 complex is located at synaptic terminals and cell bodies and regulates the synthesis of synaptic proteins in response to various stimuli (Wang and Proud, 2006). To reveal the potential molecular mechanism regulating CRS-induced synaptic damage in hippocampus of mice, we preferentially analyzed the mTORC1 signaling. As is shown in Figures 4A,B, CRS induced a noticeable decrease in the levels of p-mTOR/mTOR (*n* = 7, *p* < 0.001), in parallel with decreased phosphorylated levels of its substrates, p70S6K and 4E-BP-1 (*n* = 7, p70S6K *p* = 0.0058, p-4E-BP-1 *p* = 0.0047) (Figures 4D,E). We further investigated the upstream components of mTORC1 signaling pathways, and found a significant suppression of the phosphorylation of the Akt kinase (*n* = 7, *p* = 0.0201) (Figure 4B), which subsequently leads to the inhibition of the activity of mTORC1. It has been reported that the promoted expression of c-fos and Arc in highly active neurons triggers multiple signaling pathways, such as MAP kinase ERK signaling, contributing to the activation of one of the key transcription factors, cAMP response element-binding protein (CREB), which is integrally involved in the phenomenon of neuronal synaptic plasticity (Sun and Lin, 2016; Hou et al., 2017). The results of our Western blotting assays displayed that CRS significantly reduced the ratios of p-ERK/ERK and p-CREB/CREB (*n* = 7, p-ERK *p* = 0.0282, p-CREB *p* = 0.0364) (Figures 4C,F). Collectively, these results suggest that CRS induces synaptic dysfunction by suppressing the Akt-mTORC1 and ERK signaling pathways.

### Treatment With Rapamycin Induces Depression-like Behaviors and Synapse Loss in the Dorsal Hippocampus

**FIGURE 5:**
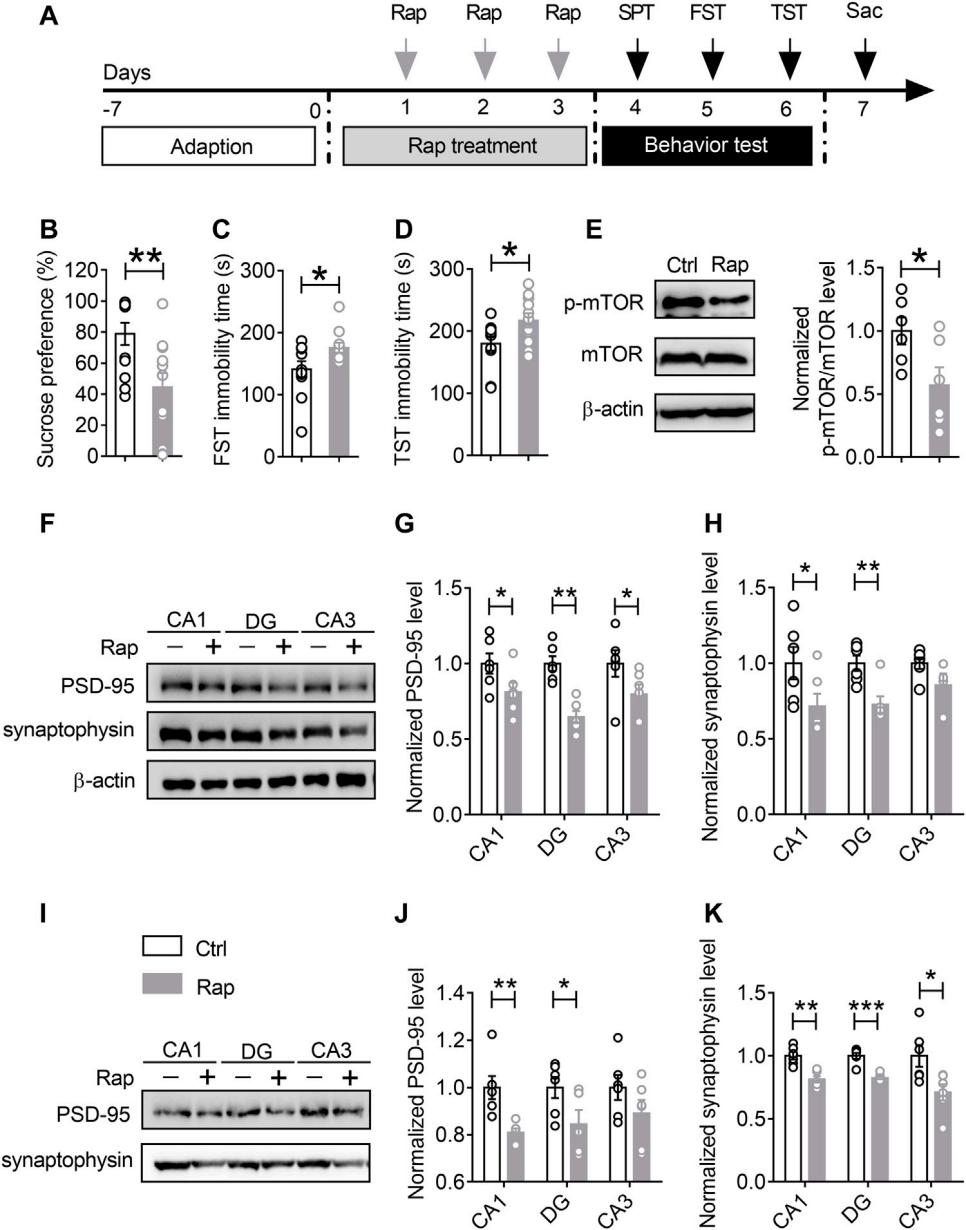
Treatment with rapamycin induces depression-like behaviors and synapse loss in the ventral hippocampus. **(A)** The schematic timeline of the experimental procedures. **(B–D)** Intracerebroventricular infusion of rapamycin simulated depression-like behaviors, including reduced sucrose preference in the SPT **(B)**, increased immobile time in the TST **(C)**, and increased floating time in the FST **(D)**. **(E–H)** Rapamycin injection decreased mTOR activity in the vHIP **(E)**, as well as the expression of PSD-95 and synaptophysin in the CA1, DG and CA3 of vHIP; representative Western blots **(F)**, statistical analysis of PSD-95 **(G)** and synaptophysin **(H)**. **(I–K)** Rapamycin treatment induced synapse loss in the CA1, DG and CA3 of vHIP; representative Western blots **(I)**, statistical analysis of PSD-95 **(J)** and synaptophysin **(K)**. Data are presented as mean ± SEM. One-way ANOVA for all. n = 11 per group for **(B–D)**; *n* = 6 per group for **(E, G,H, J,K)**. *p < 0.05, **p < 0.01, ***p < 0.001 vs. Ctrl group.

Given previous findings showing that mTORC1 signaling may be involved in synaptic protein synthesis and synapse loss in the hippocampus of mice undergoing chronic stress, we used mTORC1 inhibitor rapamycin to verify whether inhibiting the activity of mTORC1 would give rise to synaptic dysfunction and depression-like behaviors. The experimental procedures were as shown in Figure 5A. The mice were injected with rapamycin once a day for 3 consecutive days, and subsequently the behavioral tests were conducted. After that, the mice were sacrificed and the hippocampal tissues were collected after behavioral tests for Western blotting analysis. As shown in Figure 5, compared with the control mice, in the rapamycin treatment group, rapamycin injection could induce depression-like behaviors, including reduced sucrose consumption in SPT (*n* = 11, *p* = 0.0074) (Figure 5B), and increased immobility time duration in the FST (*n* = 11, *p* = 0.0382) (Figure 5C) and TST (*n* = 11, *p* = 0.0343) (Figure 5D). The results of Western blotting assays showed that mTORC1 signaling, as well as the expression levels of PSD-95 and synaptophysin in the rapamycin-treated group, were significantly decreased in both total protein samples (Figures 5E–H) and synaptic protein samples (Figures 5I–K) due to the inhibition of mTORC1 activity.

### Co-Treatment With Rapamycin Blocks the Effects of Fluoxetine

**FIGURE 6:**
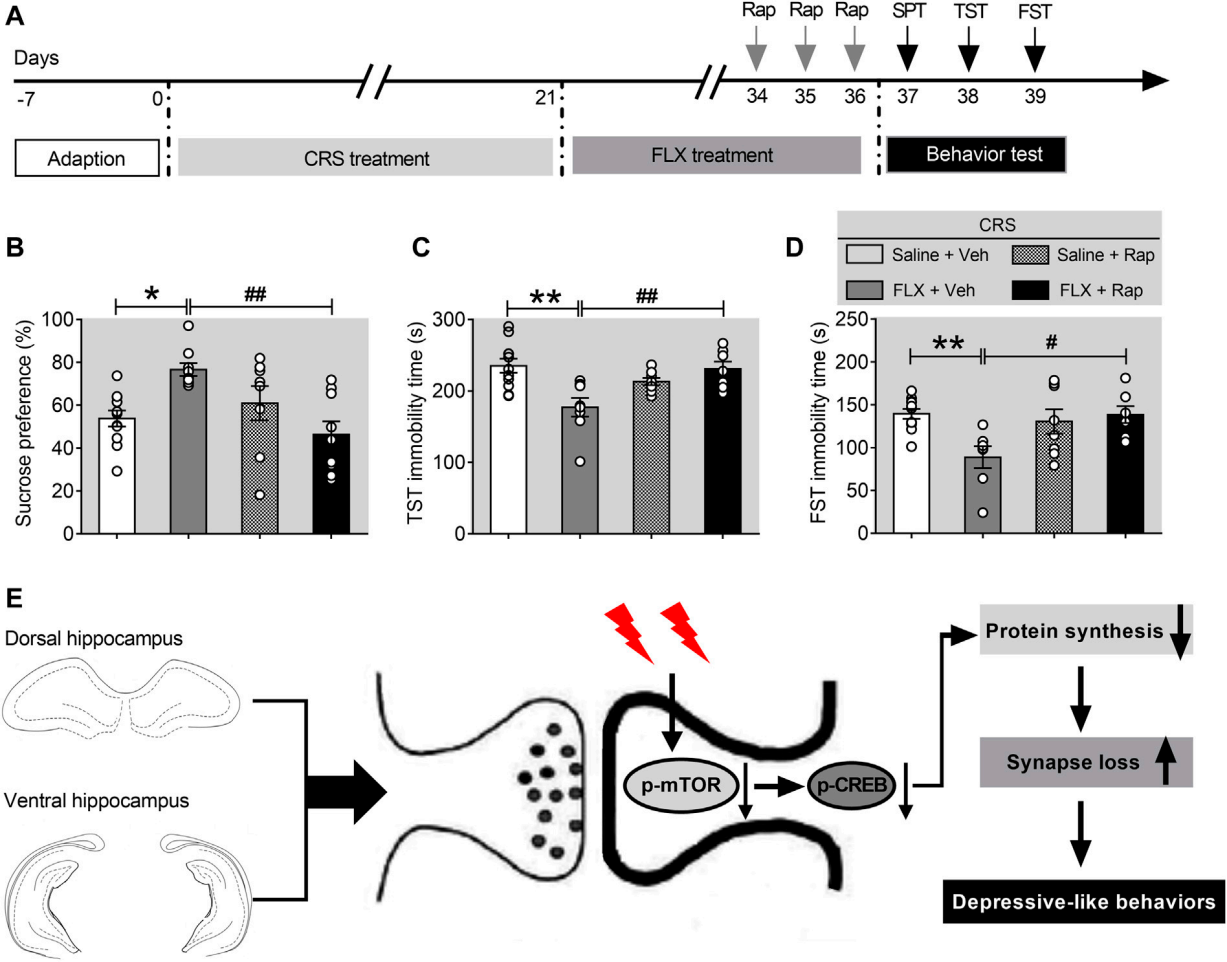
Rapamycin abolishes the effects of fluoxetine in chronically stressed mice. **(A)** The schematic timeline of the experimental procedures. **(B–D)** The antidepressant action of fluoxetine was abolished by rapamycin, including reduced sucrose preference in the SPT **(B)**, increased immobile time in the TST **(C)**, and increased floating time in the FST **(D)**. **(E)** Schematic model for the role of mTORC1 signaling pathway in mediating synapse loss induced by chronic stress. CRS suppresses the mTORC1 signaling, followed by decreased phosphorylation levels of mTORC1 signaling-associated target proteins, which triggers the downregulation of protein synthesis and the synapse loss to contribute to depression-like behaviors. Data are presented as mean ± SEM. Two-way ANOVA for all. Respectively, *n* = 11, 9, 9 and 9 per group for **(B–D)**. *p < 0.05, **p < 0.01 vs. Saline + Veh group; ^**#**^ *p* < 0.05, ^**##**^ *p* < 0.01 vs. FLX + Veh group.

Previous studies have shown that mTORC1 signaling is necessary for the antidepressant-like effects of chronic fluoxetine treatment (Xu et al., 2018). To confirm the role of mTORC1 signaling pathway in the CRS-induced depression, mice exposed to CRS for 21 days were divided into four groups, including CRS-vehicle group (CRS-Veh, *n* = 11), CRS-fluoxetine group (CRS-FLX, *n* = 11), CRS-rapamycin group (CRS-Rap, *n* = 9), and CRS-fluoxetine-rapamycin group (CRS-FLX-Rap, *n* = 9). Detailed experimental procedure was shown in Figure 6A. The results demonstrated that fluoxetine significantly improved the depression-like behaviors with increased sucrose preference (Figure 6B), as well as decreased immobility time in TST (Figure 6C) and FST (Figure 6D). However, the antidepressant effects of fluoxetine were blocked by co-treatment of rapamycin via intracerebroventricular injection. Compared with CRS-FLX group, there were significant reduction of sucrose preference (Figure 6B), and increased immobility time in TST (Figure 6C) and FST (Figure 6D) in CRS-FLX-Rap group. These results revealed that inactivation of mTORC1 signaling pathway was involved in the development of depression-like behaviors induced by chronic stress.

## Discussion

Emerging evidence demonstrates that synapse number and function are decreased in patients with MDD and bipolar disorders (BD) (Duman and Aghajanian, 2012; Kang et al., 2012). Yet, there are no direct evidence revealed for synapse loss in MDD, and the underlying mechanisms have not been identified. In this study, we used a 21-day CRS model to evaluate the synapse number and function in the hippocampus. It was found that the mRNA levels of IEGs, such as *Arc* and *Egr*, showed obvious decrease in the vHIP after CRS exposure, and the expression levels of glutamate receptors, such as GluA1, GluN2A and GluN2B, as well as the scaffolding protein PSD-95, were also decreased. To evaluate synapse loss induced by CRS in the hippocampus, we tested the presynaptic and postsynaptic markers, and observed that the expression of PSD-95 and synaptophysin significantly decreased in the dHIP and vHIP. And the expression levels of PSD-95 and synaptophysin in the presynaptic and postsynaptic fractions were also diminished. These findings indicate that CRS, a long-term stress, mediates down-regulation of the gene expression of PSD-95 and synaptophysin, and synapse loss in the hippocampus. Subsequently, we discovered that the mTORC1 signaling, which controls the synaptic protein synthesis, was impaired in the hippocampus of mice exposed to CRS. Finally, previous study has shown that chronic treatment with fluoxetine can induce synaptic protein expression by activating the mTORC1 signaling pathway, and in this study we found that intracerebroventricular infusion of rapamycin simulates depression-like behaviors and synapse loss in the hippocampus, and it abolishes the effects of fluoxetine.

Evidence from lesioning experiments has revealed that the dHIP and vHIP own different connectivity and hence, differ in their functions (Fanselow and Dong, 2010; McEown and Treit, 2010; Pacheco et al., 2017). For example, dHIP is responsible for cognitive function, including spatial learning and memory in rodents (Broadbent et al., 2004; Eckart et al., 2012), while vHIP is involved in the control of emotions and focuses on motivated behaviors through its connection with limbic system to enable behavioral flexibility (Bannerman et al., 2003; Padilla-Coreano et al., 2016). In this study, we observed that the mRNA expression of △*fos B*, the hallmark of neuronal activation, was only significantly increased in the vHIP; moreover, the mRNA levels of *Arc* and *Egr* were obviously decreased in the vHIP after CRS. These data suggest that CRS induced activation of neurons in the vHIP, and mediated a down-regulation of mRNA of *Arc* and *Egr*, which control the expression of other genes. In relation to IEGs, local protein synthesis in neuronal dendrites is critical for synaptic plasticity, and glutamate receptor and PSD-95 are involved in dendritic spine formation (Ishikawa et al., 2003; Lee et al., 2006). We first characterized the involvement of different subtypes of glutamate receptors and PSD-95 in regulating dendritic synthesis in the hippocampus, and found that PSD-95 expression was reduced in the dHIP and vHIP, as well as some subtypes of glutamate receptors, such as GluN2A and GluN2B. Interestingly, GluN1 was significantly increased in the dHIP and vHIP, which was in line with previous findings. These results suggest that the variations of IEGs mRNA may contribute to alterations in the transcription and translation of synaptic proteins. However, the signaling cascades that couple synaptic activation to dendritic protein synthesis remain elusive. There are two possible reasons for the decrease of dendritic protein levels: one is a reduction of protein synthesis, the other is the activation of protein degradation system. Consistent with the results of protein expression assays, the mRNA levels of glutamate receptors and PSD-95 were also decreased in the hippocampus after CRS. Totally, our findings suggest that vHIP may show more sensitivity to chronic stress, and synaptic protein synthesis was slowed down in the hippocampus after CRS.

Previous studies showed that mTORC1 signaling mediates synapse formation and regulates dendritic protein synthesis in neurons (Li et al., 2010; Takei and Nawa, 2014). We first detected that the phosphorylation of mTORC1 was reduced after CRS, in parallel with decreased phosphorylation levels of its substrates, p70S6K and 4E-BP-1. In addition, we also found that CRS induced a significant suppression of the phosphorylation of Akt kinase, which is an upstream activator of mTORC1. Combined with previous findings, these results suggest that mTORC1 signaling was impaired after CRS, and consequently induced a decrease of dendritic protein synthesis in hippocampal neurons. However, it was unclear whether blocking mTORC1 signaling in the hippocampus is enough to simulate depression-like behaviors. We found that intracerebroventricular infusion of rapamycin induced depression-like behaviors and synapse loss in the hippocampus. It has been reported that activation of mTORC1 signaling is required for the antidepressant actions of ketamine (Li et al., 2010; Tang et al., 2015). We also determined whether activation of mTORC1 signaling is necessary for the antidepressant actions of fluoxetine. An increase in mTORC1 signaling after fluoxetine administration showed that fluoxetine significantly activated the mTORC1 signaling which mediates synapse-associated proteins synthesis and increases synapse and spine formation. Additionally, through three well-established behavioral models of depression for mice, SPT, FST, and TST, we observed that intracerebroventricular infusion of rapamycin abolished the antidepressant effects of fluoxetine. All the above findings indicate that the mTORC1 signaling activation is sufficient to produce antidepressant effects and participates in the antidepressant action of fluoxetine.

In addition, some studies showed that rapamycin research has discrepant findings. For example, rapamycin pretreatment can prolong the antidepressant effects of ketamine (Abdallah et al., 2020), and also has been shown to exert direct antidepressant-like effects in preclinical studies following repeated treatment (Cleary et al., 2008; Autry et al., 2011; Kara et al., 2018). Two possible explanations for these divergences are the concentration of rapamycin and mode of administration. Combining previous reports and our finding, we infer that a low dose of rapamycin may have antidepressant effects and a high dose may cause depression-like behavior in mice. Additionally, systemic administration has an effect on all regions of brain and outputs antidepressant effects, while administration via local regions, such as the medial prefrontal cortex and hippocampus, mediates depression-like behavior in mice. Importantly, in order to check that the effects of rapamycin were targeted to the hippocampus, we microdissected it into CA1, DG, and CA3, and found that mTORC1 signaling was impaired and associated with synapse losses.

It is noteworthy that several questions remained to be verified. Except for principal neurons, there are many other types of neurons in the hippocampus (Szilágyi et al., 2011; Wheeler et al., 2015), it would be appropriate to investigate which type of neurons are damaged in the hippocampus after CRS. Another interesting observation in our experiment is that fluoxetine increased the activity of mTORC1 in the hippocampus, but how fluoxetine affects the activity of mTORC1? Fluoxetine, a selective serotonin reuptake inhibitor, regulates serotonin at synapse, and previous studies show that serotonin mediates the expression of brain-derived neurotrophic factor (BDNF) (Jiang et al., 2016), which induces the phosphorylation and activation of cAMP-response element binding protein (CREB) through binding its receptor tyrosine kinase B (TrkB) (Kim et al., 2012; Jiang et al., 2016; Bai et al., 2019). BDNF-TrkB signaling initiates the downstream signaling cascades (MAPK/ERK and PI_3_K/Akt pathways) and hence, activates the mTORC1 signaling pathway and produces an antidepressant effect (Abelaira et al., 2014). However, this conjecture needs to be investigated by inhibiting the BDNF signaling pathway, for example, BDNF-shRNA lentivirus or TrkB antagonists. All these experiments will be performed in our further study.

## Conclusion

In summary, our present study provides evidence that suppression of mTORC1 signaling pathway plays an important role in mediating synapse loss induced by chronic stress, and mTORC1 signaling has part in the behavioral effects of antidepressant treatment. These findings also suggest that compounds which upregulate the mTORC1 signaling in the hippocampus may be effective for the treatment of depression.

## Data Availability

The original contributions presented in the study are included in the article/Supplementary Material, further inquiries can be directed to the corresponding authors.

## Abbreviations

AMPARs, AMPA-type glutamate receptors; CREB, cAMP response element-binding protein; CRS, chronic restraint stress; Ctrl, control; DG, dentate gyrus; dHIP, dorsal hippocampus; ERK, extracellular signal-regulated kinase; FLX, fluoxetine; FST, forced swim test; mTORC1, mechanistic target of rapamycin complex 1; NMDARs, NMDA-type glutamate receptors; PSD-95, postsynaptic density protein 95; SPT, sucrose preference test; TST, tail suspension test; Veh, vehicle; vHIP, ventral hippocampus.
