# Supplementary material for: mTORC1 Signaling Pathway Mediates Chronic Stress-Induced Synapse Loss in the Hippocampus
Source: Front Pharmacol. 2021 Dec 20;12:801234. doi: 10.3389/fphar.2021.801234 (PMC8722735; doi:10.3389/fphar.2021.801234)
Supplement: Supplementary file 1 [file DataSheet1.docx]

**Supplementary Material**

**mTORC1 signaling pathway mediates chronic stress-induced synapse loss in the hippocampus**

Running title: Synapse loss and mTORC1 signaling

Yu-Fei Luo, Xiao-Xia Ye, Ying-Zhao Fang, Meng-Die Li, Zhi-Xuan Xia, Jian-Min Liu, Xiao-Shan Lin, Zhen Huang, Xiao-Qian Zhu, Jun-Jie Huang, Dong-Lin Tan, Yu-Fei Zhang, Hai-Ping Liu, Jun Zhou, Zu-Cheng Shen

**Supplementary Figures**


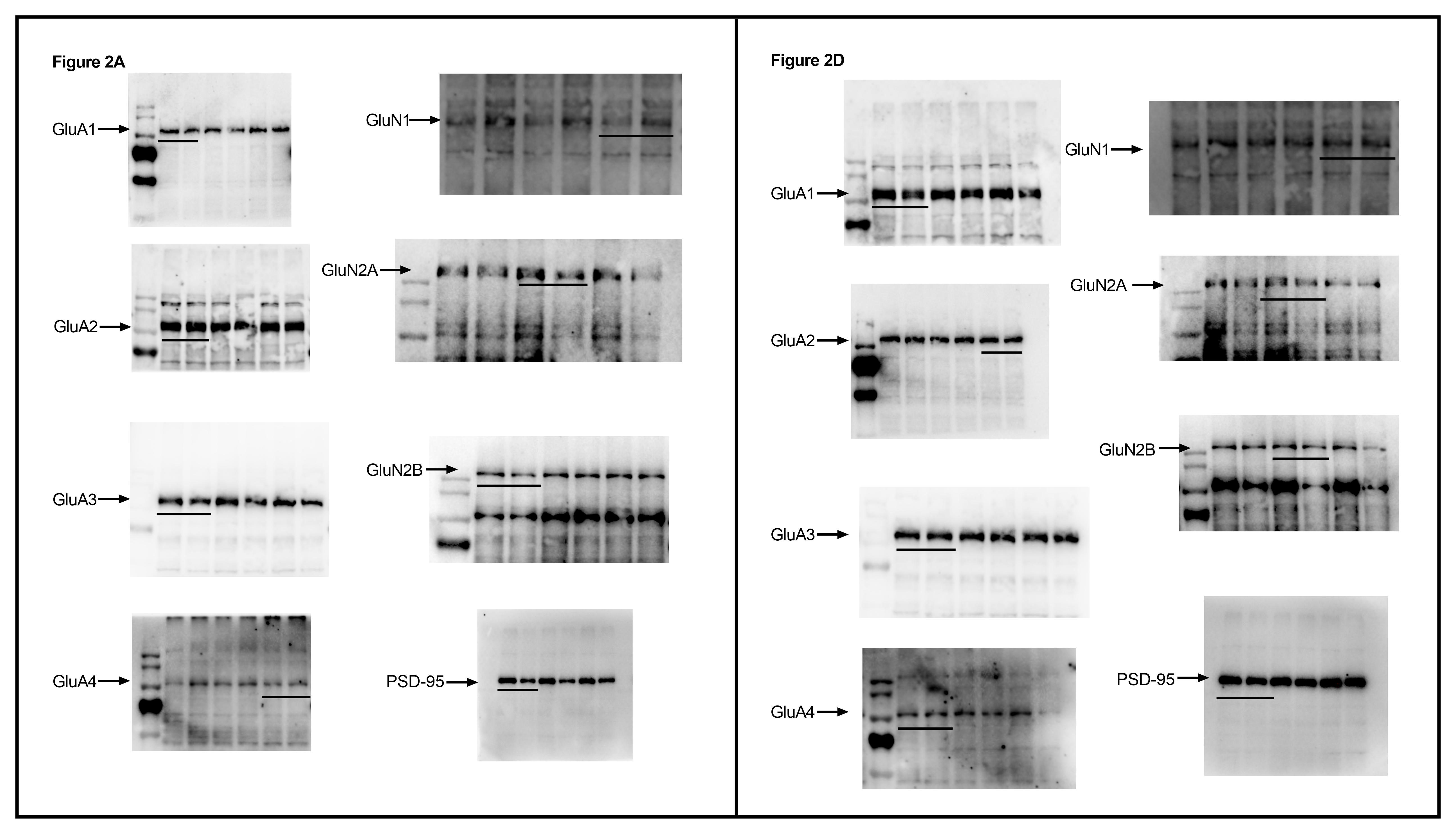


**Figure S1. Full original images of Western blotting assays for Figure 2.**


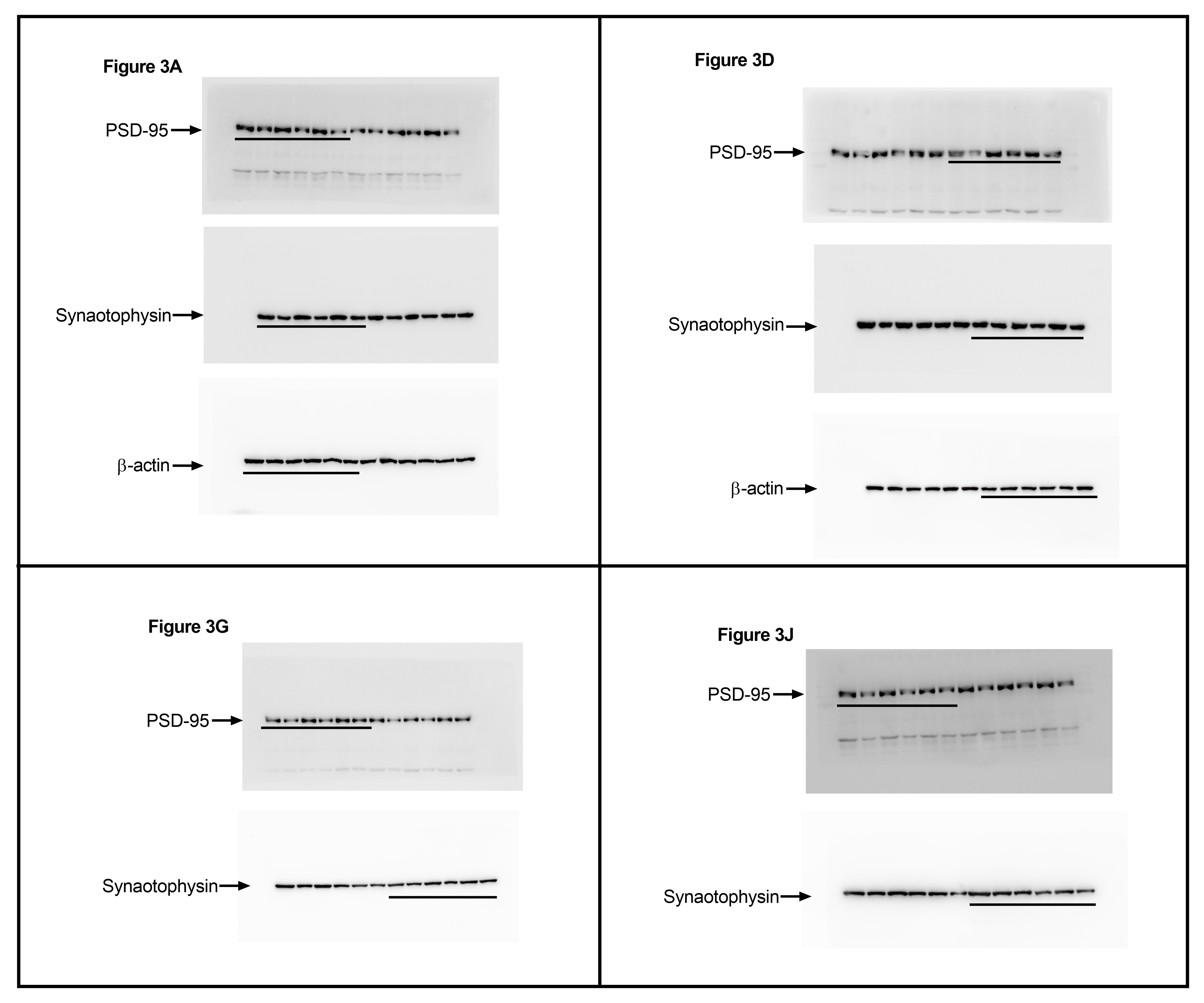


**Figure S2. Full original images of Western blotting assays for Figure 3.**


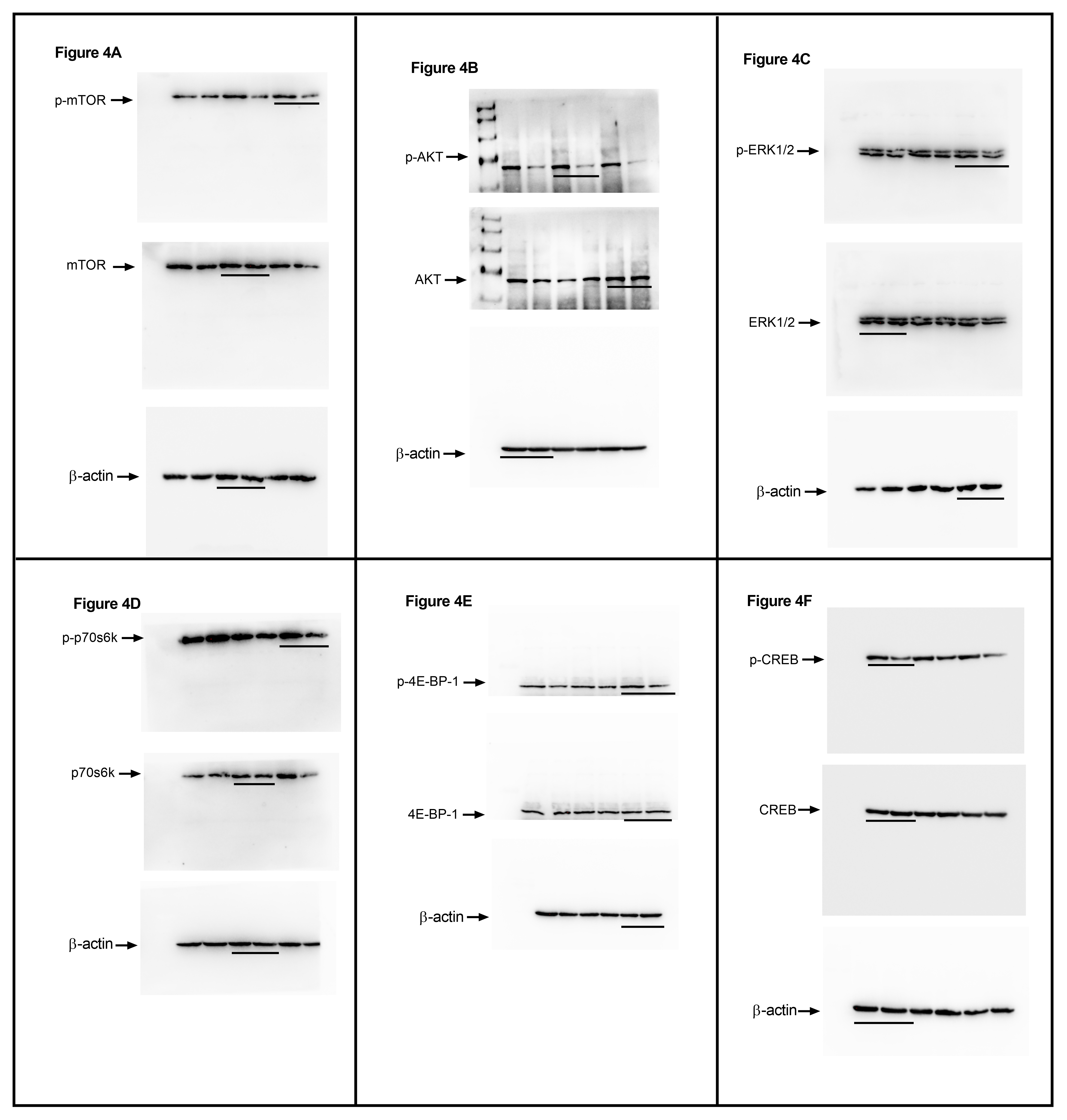


**Figure S3. Full original images of Western blotting assays for Figure 4.**


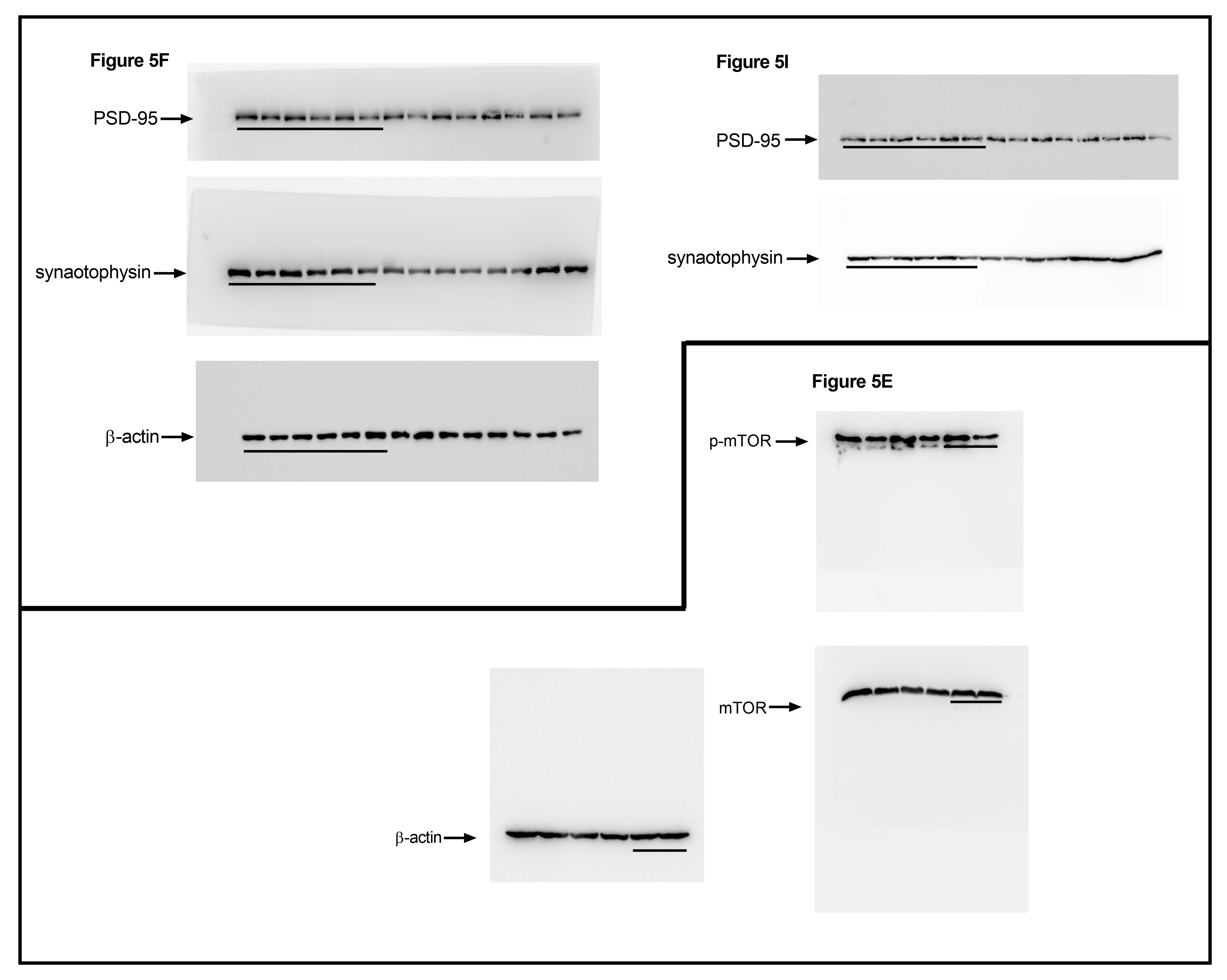


**Figure S4. Full original images of Western blotting assays for Figure 5.**

**Supplementary Tables**

**Table S1. Information of antibodies used in this study.**

| **Antigen protein (kDa)** | **Host** | **Dilution** | **Catalog** | **Manufacturer** |
| --- | --- | --- | --- | --- |
| GluA1 (102) | Rabbit | 1/1000 | ab31232 | Abcam, UK |
| GluA2 (99) | Rabbit | 1/1000 | ab20673 | Abcam, UK |
| GluA3 (95) | Rabbit | 1/1000 | ab40845 | Abcam, UK |
| GluA4 (102) | Rabbit | 1/1000 | ab53088 | Abcam, UK |
| PSD-95 (95) | Rabbit | 1/1000 | #3409 | CST, USA |
| GluN1 (120) | Rabbit | 1/1000 | #5704 | CST, USA |
| GluN2A (165) | Rabbit | 1/1000 | ab124913 | Abcam, UK |
| GluN2B (166) | Rabbit | 1/1000 | ab65783 | Abcam, UK |
| synaptophysin (34) | Rabbit | 1/1000 | ab165659 | Abcam, UK |
| ERK1/2 (44) | Rabbit | 1/1000 | #4695 | CST, USA |
| p-ERK1/2 (43) | Rabbit | 1/1000 | #4370 | CST, USA |
| mTOR (289) | Rabbit | 1/1000 | ab134903 | Abcam, UK |
| p-mTOR (289) | Rabbit | 1/1000 | ab109268 | Abcam, UK |
| AKT (56) | Rabbit | 1/1000 | #4685 | CST, USA |
| p-AKT (56) | Rabbit | 1/1000 | #4060 | CST, USA |
| p70s6k (53) | Rabbit | 1/1000 | ab184556 | Abcam, UK |
| p-p70s6k (53) | Rabbit | 1/1000 | #97596 | CST, USA |
| 4E-BP-1 (13) | Rabbit | 1/1000 | #9644 | CST, USA |
| p-4E-BP-1 (13) | Rabbit | 1/1000 | #9456 | CST, USA |
| CREB (37) | Rabbit | 1/1000 | ab32515 | Abcam, UK |
| p-CREB (37) | Rabbit | 1/1000 | ab32096 | Abcam, UK |
| β-actin (42) | Mouse | 1/3000 | sc-47778 | Santa Cruz, USA |

**Table S2. Oligonucleotide primers used for mRNA real-time qPCR.**

| **Gene** | **Sense primer (5’ to 3’)** | **Antisense primer (5’ to 3’)** |
| --- | --- | --- |
| c-fos | GTTTCAACGCGGACTACGAG | GGCACTAGAGACGGACAGAT |
| Delta-fos | AGGCAGAGCTGGAGTCGGAGAT | GCCGAGGACTTGAACTTCACTCG |
| Arc | GCATCTGTTGACCGAAGTGTCC | GCACCCAAGACTGGTATTGCTG |
| Egr | GAGGAAGTTTGCCAGGAGTG | GAGTAGGAAGTGGGCACAGG |
| PSD-95 | CCCCCAGACATCACAACCTC | CAGGGGAGTAGCGCCGAGG |
| GluN1 | TCATCTCTAGCCAGGTCTACG | CAGAGTAGATGGACATTCGGG |
| GluN2A | GCACCAGTACATGACCAGATTC | ACCAGTTTACAGCCTTCATCC |
| GluN2B | TTCATGGGTGTCTGTTCTGG | GGATGTTGGAGTGGGTGTTG |
| GluA1 | GGAAGGAAGGGAGGAAGGAAAG | GGAGAACTGGGAACAGAAACGG |
| GluA2 | AGGCAGAGCTGGAGTCGGAGAT | GCCGAGGACTTGAACTTCACTCG |
| GluA3 | AACGCCTGTAAACCTTGCAGT | AGTCCTTGGCTCCACATTCC |
| GluA4 | CCAGGGCAGAGGCGAAG | CGTTTTCTCCCACACTCCCA |
| GAPDH | ATGGTGAAGGTCGGTGTG | CATTCTCGGCCTTGACTG |
